# Epidemiology of childhood blindness: A community-based study in Bangladesh

**DOI:** 10.1371/journal.pone.0211991

**Published:** 2019-06-07

**Authors:** A. H. M. Enayet Hussain, Junnatul Ferdoush, Saidur Rahman Mashreky, A. K. M. Fazlur Rahman, Nahid Ferdausi, Koustuv Dalal

**Affiliations:** 1 Directorate General of Health Services, Dhaka, Bangladesh; 2 Centre for Injury Prevention and Research Bangladesh, Dhaka, Bangladesh; 3 Bangladesh University of Health Sciences, Dhaka, Bangladesh; 4 National Institute of Ophthalmology, Dhaka, Bangladesh; 5 School of Health and Education, University of Skövde, Skovde, Sweden; 6 Higher School of Public Health, Al-Farabi Kazakh National University, Almaty, Kazakhstan; Anglia Ruskin University, UNITED KINGDOM

## Abstract

This study aimed to investigate the prevalence and causes of childhood blindness in a rural area of Bangladesh. We adopted a cross-sectional quantitative study design for this study, which was performed in three unions (sub-districts) located in Raiganj Upazila of the Sirajganj district in Bangladesh. Using a validated tool, a screening program was conducted at the household level. After initial screening, a team of ophthalmologists confirmed the diagnoses by clinical examinations. The prevalence of childhood blindness was observed to be 6.3 per 10,000 children, whereas the rate of uniocular blindness was 4.8 per 10,000 children. Congenital problems were the major causes of both uniocular and binocular blindness (uniocular blindness: 84% and binocular blindness: 92%). The whole globe was the site responsible for binocular blindness (28.0%, 95% confidence interval [CI]: 13.1, 47.7), whereas the cornea was responsible for uniocular blindness (57.8%, 95% CI: 35.3, 78.1). Childhood blindness is a public health problem in Bangladesh and is highly prevalent, regardless of sex. The major causes of childhood blindness are congenital.

## Introduction

Globally, an estimated 36 million people live with blindness [1]. In terms of the prevalence of blindness by age distribution, around 1.4 million children aged 0–14 years are currently living with blindness, whereas approximately 17.5 million are at a risk of developing low vision [2]. The estimated burden associated with blindness among children is 70 million blind person years [3]. Although the actual number of blind children is much lower than that of blind adults, the number of blind years resulting from the blindness is alarmingly high in children, and this has an immense social and economic impact [4–6].

The magnitude and causes of visual impairment and blindness vary by region, owing to socio-developmental diversification [4]. Analyses of global data showed that around 90% of blind people reside in developing countries [7]. Few population-based studies in recent times have investigated the prevalence as well as factors responsible for childhood blindness in the context of developing countries. However, it has been observed that the burden of childhood blindness is higher in the African and Asian regions, predominantly because of inaccessibility to primary healthcare services [5,8,9].

A majority of the causes of childhood blindness are avoidable even in the minimal resource settings of developing countries [7]. In Bangladesh, a lower middle-income country, the conditions are significantly worse in the rural areas, in which there are enormous issues pertaining to the delivery of healthcare [10]. Owing to socioeconomic and cultural constraints, and the medical poverty trap settings, it could be assumed that Bangladesh, especially its rural areas, has poor eye care facilities [11]. Over the last few years, a number of initiatives have been commenced with the aim of achieving the VISION 2020 goal [12,13]. However, there is further scope for improvement. Moreover, few existing studies have investigated the burden and capacity of the healthcare system in Bangladesh in terms of childhood blindness [14,15]. Very little information is explored and documented about epidemiology of childhood blindness. However, for designing a prevention strategy comprehensive epidemiological information is crucial. Considering the gap, this study was designed to explore and document the prevalence and causes of childhood blindness in a rural area of Bangladesh.

## Materials and methods

### Study design

A cross-sectional quantitative design was adopted for this study, which was conducted from January to April 2017 in the three unions of Raiganj, a sub-district (*upazila*) of the Sirajganj District in the Rajshahi Division of northern Bangladesh.

### Study population

The whole area of the targeted *upazila* is 259.74 km^2^, with an established population density of 223 per sq. km. Approximately 317,666 people reside in this *upazila*; 51.1% of the population is male and 49.9% is female [16]. The *upazila* comprises nine unions. A union is the lowest administrative unit and comprises a population of 20,000–50,000 people. A survey was conducted in three of the nine unions in which the Center for Injury Prevention and Research Bangladesh (CIPRB) maintains an injury and demographic surveillance system. The entire population of the three unions was included in our surveillance system; it included 31,971 households with a population of 147,072 people, and the total number of children aged ≤15 years was 39,351. The survey was conducted among the entire population of children in the indicated study areas. The target population comprised those aged 15 years or younger, according to the operational definition.

### Data collection

A household-level screening was conducted for the identification of suspected childhood blindness, visual impairment, and other ocular morbidities. In the screening, data collectors conducted a face-to-face interview along with some simple examinations using a validated structured questionnaire with pictorial charts S1–S3 Files. The instrument used in this study was previously piloted in the Sylhet Division of Bangladesh. Each data collector was provided training on the measurement of visual acuity using an age-specific vision chart and the performance of a basic ocular examination. A day-long comprehensive training session was provided by a team of both researchers and ophthalmologists. Respondents’ socio-demographic information was collected from the CIPRB surveillance system database. All individuals in the surveillance area have their own unique identification number. After collection, specific information was merged with the required socio-demographic variables in the existing population database.

A total of 18 field workers (three field supervisors and 15 data collectors) conducted the survey. If any household member was not available during the data collection period, field workers collected their contact number and visited them again based on convenience. The data collectors identified 570 suspected cases of blindness, visual impairment, and other ocular morbidities.

All the children in whom the aforementioned suspicions were raised, were then invited to visit the eye camp for the confirmation of disease presence and provision of further management advice. The field workers invited all the parents/caregivers of these children through telephonic conversations; for those who could not be reached through the telephone, a personal meeting was conducted to ensure the children’s presence at the eye camp. All families were financially compensated for the transportation costs associated with reaching the eye camp. A day-long eye camp was conducted in three phases to examine all the suspected cases. The team comprised five ophthalmologists, two health assistants and five supporting staff members.

**Fig 1:**
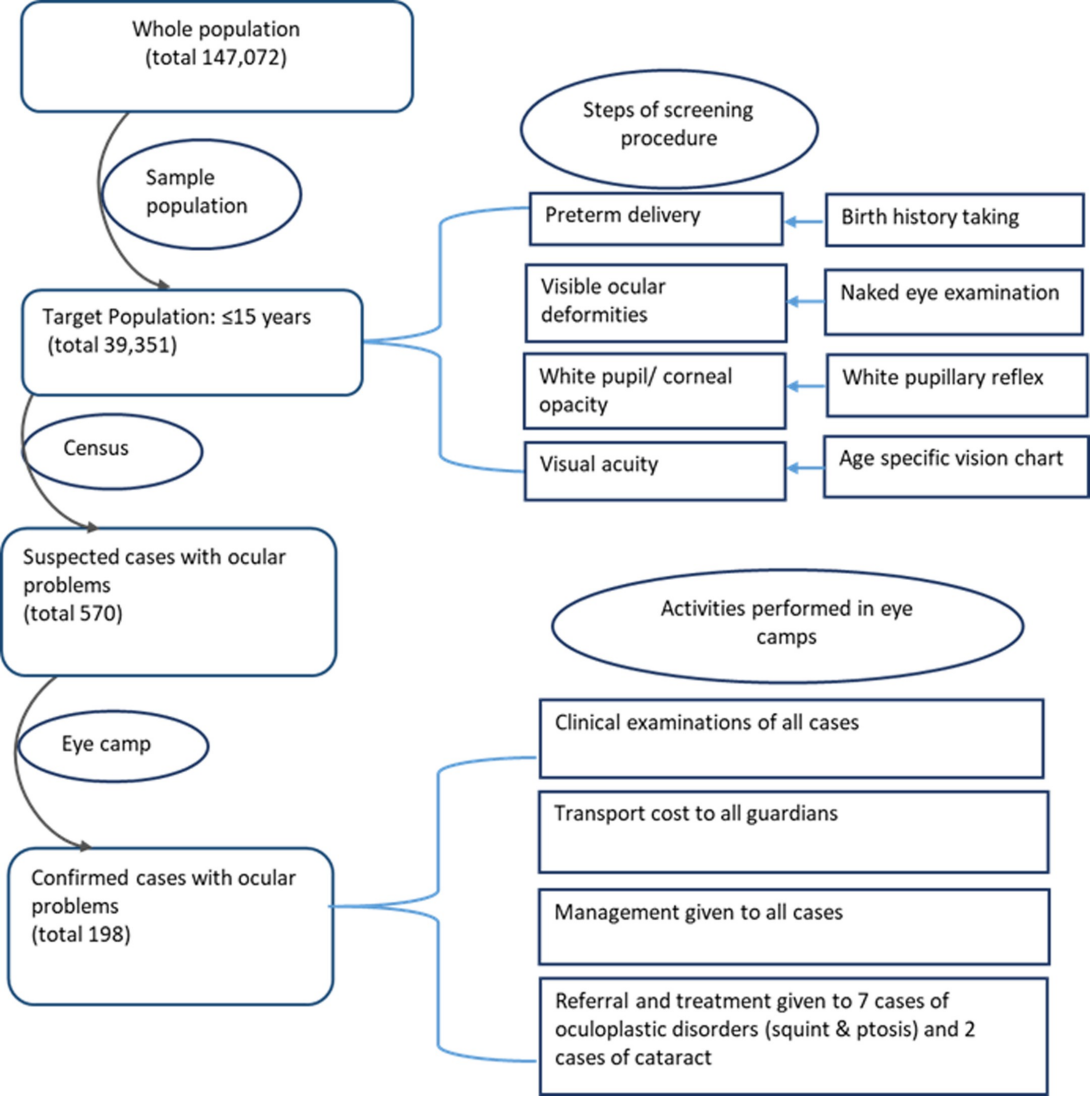
Methodological protocol of this study.

All children were examined based on standard clinical guidelines, after written informed consent for participation was provided by their legal guardian. Ophthalmologists used a Snellen chart, slit lamp, retinoscope, direct ophthalmoscope and indirect ophthalmoscope to confirm blindness, visual impairments, and ocular morbidities. Of the 570 cases that were screened, finally 198 cases of blindness and eye-related morbidities were confirmed by the team. The ophthalmologists maintained records pertaining to the principal reason as well as the reasons contributing to blindness/visual impairment. Of the confirmed cases, a total of nine were referred to specialized hospitals for the performance of indicative surgery. Other confirmed cases were referred to different healthcare facilities for the reception of definitive treatment (Fig 1).

### Statistical analysis

Descriptive analyses were performed to describe the population by sex, age, and socioeconomic status. Similar analyses were conducted to estimate the prevalence of childhood blindness and its associated risk factors. The prevalence of all types of blindness was calculated per 10,000 children with its 95% confidence interval (CI). The prevalence of childhood blindness was described by sex, age, wealth index, and classification of blindness. For the purpose of analysis, children were categorized into the “under five” and “5–15” years age groups, whereas the wealth index was categorized as “poor,” “middle,” and “rich.” Bivariate analyses were performed to analyze the relationship between childhood blindness and independent variables such as sex, age group, wealth index, and the classification of blindness. All statistical analyses were performed using SPSS version 24 (IBM Corp., Armonk, NY, USA).

### Operational definitions

#### Children

In this study, those aged 15 years or younger were considered children.

#### Blindness

The definition of blindness, as per the International Classification of Diseases, 10^th^ edition (ICD-10), was considered for this study. Blindness was defined as a corrected visual acuity lower than 3/60 in the better eye or a central field lower than 10 degrees [17]. The World Health Organization’s standard anatomical and etiological classification was used in this study [18].

#### Binocular and uniocular blindness

The ICD-10 standard definitions of binocular and mono/uniocular blindness were considered for this study [17].

#### Reversible blindness

Temporary vision loss that could be treated through surgical intervention was termed “reversible blindness”.

#### Irreversible blindness

This was defined as vision loss that could not be restored either through medical or surgical interventions.

#### Hereditary

The ocular problems those were present since birth.

#### Acquired

The visual problems those were developed after birth.

#### Wealth index

The wealth index was categorized using some selective assets of the household, such as: the unit of land ownership; availability of amenities (i.e., electricity, refrigerator, television, radio, bicycle, motorcycle, wardrobe, table, chair, clock, bed, sewing machine, mobile, car, water, and toilet); fuels used daily; and materials used for building the roof, floor, and walls of the household.

### Ethical consideration

The ethical clearance of this study was received from the Ethical Review Committee of the CIPRB. We obtained written consent from all the participants’ legal guardians during the screening and ophthalmologist visit.

## Results

In this study, the proportions of male (51%) and female (49%) children were similar. The majority of the children were in the 5–15 years age group (70%), and the remaining were aged 5 years or less. In terms of the three wealth index categories, most of the children belonged to the ‘poor’ category (50%) (Table 1).

**Table 1:** Population characteristics (N = 39,351).

Variables	Frequency	Percentage
**Sex**		
Male	20211	51.3
Female	19140	48.6
**Age (in years)**		
< 5 years	11806	30.0
5 to 15 years	27545	70.0
**Wealth index**		
Poor	19678	50.0
Middle-class	9843	25.0
Rich	9830	25.0

The prevalence of binocular blindness in our study population was 6.3 per 10,000 children, whereas that of uniocular blindness was 4.8 per 10, 000 children. Both uniocular and binocular blindness were more prevalent among boys than girls; however, the difference was not statistically significant. A significantly higher rate of binocular blindness was observed among those aged under 5 years; the rates were 19.4 (95% CI: 12.6–28.7) and 0.7 (95% CI: 0.1–2.3) in children aged under 5 years and those aged 5 years and above, respectively. Binocular blindness showed a higher prevalence among those with the “poor” wealth index than those who were “rich;” however, the difference was not statistically significant. The prevalence rates of binocular blindness were 8.6 (95% CI: 5.2–13.5) and 2.0 (95% CI: 0.3–6.7) among the “rich” and “poor” children, respectively. On the other hand, a higher prevalence rate of uniocular blindness was observed among those with the “middle class” wealth index; the rate was 8.1 (95% CI: 3.7–15.4) (Table 2).

**Table 2:** Distribution of binocular and uniocular blindness, by age, sex, and socioeconomic status.

Variable	Binocular blindness		Total	Uniocular blindness		Total
	Reversible	Irreversible		Reversible	Irreversible	
	Rate per 10,000 children (95% CI)					
Sex						
Male (n = 20211)	0.9 (0.1,3.2)	6.4 (3.5,10.7)	7.4 (4.3,11.9)	4.9 (2.5,8.7)	0.4 (0.03,2.4)	5.4 (2.8,9.4)
Female (19140)	3.1 (1.2,6.5)	2.0 (0.6,5.0)	5.2 (2.6,9.3)	2.0 (0.6,5.0)	2.0 (0.6,5.0)	4.1 (1.9,7.9)
Age group						
Under 5 years (n = 11806)	5.9 (2.5,11.7)	13.5 (8.0,21.5)	19.4 (12.6,28.7)	5.0 (2.0,10.5)	2.5 (0.6,6.9)	7.6 (3.7,13.9)
5–15 years (n = 27545)	0.3 (0.04,1.7)	0.3 (0.04,1.7)	0.7 (0.1,2.3)	2.9 (1.3,5.5)	0.7 (0.1,2.3)	3.6 (1.8,6.4)
Wealth index						
Poor (n = 19678)	2.5 (0.9,5.6)	6.0 (3.2,10.3)	8.6 (5.2,13.5)	1.5 (0.4,4.1)	1.5 (0.4,4.1)	3.0 (1.1,6.3)
Middle class (n = 9843)	3.0 (0.8,8.2)	4.0 (1.3,9.8)	7.1 (3.0,14.0)	7.1 (3.0,14.0)	1.0 (0.03,5.0)	8.1 (3.7,15.4)
Rich (n = 9830)	0	2.0 (0.3,6.7)	2.0 (0.3,6.7)	4.0 (1.3,9.8)	1.0 (0.03,5.0)	5.0 (1.8,11.2)
Total (n = 39351)	2.0 (0.9,3.8)	4.3 (2.5,6.7)	6.3 (4.2,9.2)	3.5 (2.0,5.8)	1.2 (0.4,2.8)	4.8 (2.9,7.3)

CI, confidence interval

In a majority of the uniocular and binocular blindness cases (binocular blindness: 92% and uniocular blindness: 84%), the cause was of a congenital nature. For binocular blindness, the whole globe was predominantly the site of structural origination (28.0% [95% CI: 13.1–47.7]), while the cornea was the site responsible for uniocular blindness (57.8% [95% CI: 35.3–78.1]). The second most prevalent anatomical site was Central Nervous System (CNS) for binocular blindness with the proportion of 20.0% (95% CI: 7.7–38.9), whereas retina and optic nerve were found for uniocular blindness with the proportions of (15.7% [95% CI: 4.1–37.2]) and (15.7% [95% CI: 4.1–37.2]) (Table 3).

**Table 3:** Distribution of binocular blindness and uniocular blindness by anatomical and etiological classification.

	Binocular blindness (N = 25)	Uniocular blindness (N = 19)
	**Proportion (95% CI)**	
**Main site**		
Whole globe	28.0 (13.1, 47.7)	0
Cornea	4.0 (0.1, 18.1)	57.8 (35.3, 78.1)
Lens	16.0 (5.2, 34.2)	0
Uvea	0	0
Retina	12.0 (3.1, 29.2)	15.7 (4.1, 37.2)
Optic nerve	8.0 (1.3, 24.0)	15.7 (4.1, 37.2)
Glaucoma	4.0 (0.1, 18.1)	5.2 (0.2, 23.3)
CNS	20.0 (7.7, 38.9)	5.2 (0.2, 23.3)
Other (angle of anterior chamber)	8.0 (1.3, 24.0)	0
**Etiological**		
Hereditary	92.0 (76.0, 98.6)	84.2 (62.7, 95.8)
Acquired	8.0 (1.3, 24.0)	15.7 (4.1, 37.2)

CI, confidence interval

## Discussion

The household-level survey employed in this study is the first of its kind to be conducted in a rural community of Bangladesh, in which the prevalence of childhood blindness was estimated. We found that the prevalence rate of childhood blindness was 6.3 per 10,000 children. Through the extrapolation of this number to the national target group, we found that around 35,000 children in Bangladesh are currently living with blindness. In 2007, Muhit et al. claimed that the estimated number of children with blindness/severe visual impairments was 26,000 [14]. However, the present study and that by Muhit et al. followed different methodologies; while our study performed screening through a house-to-house community-based survey, the latter followed a combination of special child inclusion from the institution and the capturing of blindness cases through the key informant method. Another study reported that approximately 40,000 children in Bangladesh are living with blindness [15]. It must be noted that our study data are applicable to rural settings only, and further rigorous research is warranted in both the rural and urban context.

This is the first study to have estimated the prevalence and causes of uniocular reversible and irreversible blindness among children in Bangladesh. No previous study in Bangladesh has examined the burden and etiology associated with uniocular blindness. A community-based study in Oman showed that the occurrence rate of uniocular blindness is 9 per 10,000 children [19]. The estimated number of blindness cases is likely to double if the contributory number of uniocular blindness is combined with binocular blindness [19]. This information may prove helpful to health policymakers in priority-setting. Cases of persistent untreated unilateral blindness, particularly those caused by ocular trauma, may eventually progress to blindness [20]. Although the prevalence of uniocular blindness does not directly contribute to the burden of total blindness, early diagnosis and the treatment of reversible uniocular blindness will eventually contribute to reducing the burden of total blindness. In this context, the burden of uniocular blindness is also an emerging public health concern in Bangladesh.

A 2003 study conducted in Andhra Pradesh, India, found that a majority of children with blindness were female [21]. In our study, the prevalence of blindness was higher among boys; however, the difference was not statistically significant. While many studies have shown that blindness is much more prevalent among those with a poor financial status [22], the present study’s findings demonstrated no statistically significant differences in the prevalence between those with a “poor” and “rich” wealth index.

Congenital causes were the most common reasons for both uniocular and binocular childhood blindness in our study. Additionally, the whole globe was the site predominantly responsible for binocular blindness, whereas the cornea was predominantly responsible for uniocular blindness. A previous nationwide study stated that the lens is the most commonly observed anatomical site in unoperated cataract cases [23]. Improved maternal and childcare initiatives taken may help reduce the national burden of cataract in children in Bangladesh. A study conducted among school-going children in China also found that the whole globe was the anatomical site responsible for childhood blindness [24]. Evidences showed that glaucoma in children is a rare visual problem [25–28] which is also consistent with the present study’s findings. Though developed countries have tackled the burden of childhood glaucoma with their advanced medical technologies, developing countries are still facing an enormous burden [28]. Data found that the traumatic eye injury is one of the most common cause of uniocular blindness for children of all ages [29], but in this study, we did not aim to explore this particular field. Vitamin A deficiency has been shown to be a major cause of childhood vision loss [23]; nevertheless, in our study, this deficiency was not identified as a leading cause of childhood blindness, owing probably to the continuous spectacular progress in the healthcare and nutrition sectors in Bangladesh. The National Vitamin-A plus campaign is a successful program held each year in Bangladesh, which contributes tremendously to the control of night blindness development [30].

### Strengths and limitations

In this study, screening for childhood blindness at a community level was performed through a house-to-house survey, as described by Nirmalan et al. in South India [31]. It is suggested that the use of the community door-to-door approach is credible in the determination of the extent of childhood blindness, as it is associated with lower rates of drop-outs. Moreover, this survey was conducted within an established Health and Demographic Surveillance System, which covered a population greater than 150,000 people. All the children aged 15 years and younger in the study area were included in our study, minimizing sampling errors. A group of senior ophthalmologists were involved in the diagnostic procedure, while an experienced ophthalmologist led the diagnostic team; this may have contributed to minimizing errors related to disease misclassification. The study was conducted in a rural area of Bangladesh; therefore, its findings may not be applicable to children living in the urban areas of the country. Additionally, the relatively small sample size of our study should be considered in the interpretation of its findings.

## Conclusions

Childhood blindness is a significant public health concern in Bangladesh and is highly prevalent, regardless of sex. Young children are found more sufferer from blindness. A majority of the childhood blindness cases have congenital causes.

This study’s findings indicate that necessary measures need to be taken to reduce the prevalence of childhood blindness in Bangladesh. The estimated prevalence obtained from this study can be used to conduct further research in this particular domain in different geographical locations in Bangladesh inclusion of urban areas. Early diagnosis of childhood visual impairment is the key to early intervention, and an important part of preventing childhood blindness. Raising social awareness on reversible childhood blindness is important, as early identification and treatment can reverse the condition. Strategies need be developed, aimed at proper strengthening of the health system for the prevention of childhood blindness in Bangladesh.

## Supporting information

S1 File Original survey questionnaire. (DOCX)

S2 File Survey questionnaire English version. (DOCX)

S3 File Survey pictorial flip cards. (PDF)

S4 File Original dataset of this study. (SAV)
