# Supplementary material for: Epidemiology of childhood blindness: A community-based study in Bangladesh
Source: PLoS One. 2019 Jun 7;14(6):e0211991. doi: 10.1371/journal.pone.0211991 (PMC6555501; doi:10.1371/journal.pone.0211991)
Supplement: S1 File — (DOCX) [file pone.0211991.s001.docx]

লিখিত সম্মতিপত্র

শিশুর মা-বাবা অথবা যত্নগ্রহণকারী জন্য

**আমার নাম..................... । আমরা একটি গবেষণা পরিচালনা করছি এবং আমাদের গবেষণার উদ্দেশ্য হচ্ছে** ১৫ বছর এবং এর নিচের বয়সী শিশুদের চোখের সমস্যা এবং অন্ধত্ব এর হার এবং কারণগুলো খুঁজে বের করা এবং সেই পরিপ্রেক্ষিতে শিশু অন্ধত্ব প্রতিরোধমূলক একটি ফ্রেমওয়ার্ক প্রতিষ্ঠা করা। আপনার শিশুকে আমরা বাছাই করেছি কারণ তার বয়স ১৫ বছরের মধ্যে। আমরা এটি নিশ্চিত করতে চাই যে এই গবেষণায় অংশ গ্রহণের পূর্বে আপনি আমাদের উদ্দেশ্য এবং আপনার দায়িত্ব সম্পর্কে বুঝতে পেরেছেন। আপনি যদি আমার কোন কথা না বুঝতে পারেন আমাকে জিজ্ঞেস করতে পারবেন।

**আমি আপনাকে আপনার শিশু সম্পর্কে কিছু প্রশ্ন করবো, পাশাপাশি আমি একটি টর্চ দিয়ে তার চোখ দেখবো এবং বয়স উপযোগী টুল (টর্চ, রঙ্গীন মিষ্টি জাতীয় জিনিস এবং চার্ট) এর মাধ্যমে আপনার শিশুর চোখের তীক্ষ্ণতা পরীক্ষা করবো যার জন্য আমার ১৫-২০ মিনিট সময় লাগবে। আপনার সকল উত্তর এবং আপনার শিশুর চক্ষু পরীক্ষার ফলাফল গোপন রাখা হবে এবং গবেষনা দলের বাহিরে কারো সাথেই এই বিষয়ে আলোচনা করা হবে না। আপনার ব্যক্তিগত তথ্য গবেষণা ব্যতিত অন্য কোন কাজে ব্যবহার করা হবে না।**

**আপনার অংশগ্রহন হবে স্বেচ্ছা মূলক এবং আপনি কোনো প্রশ্নের উত্তর দিতে স্বাচ্ছন্দ্য বোধ না করলে আপনাকে উত্তর দিতে হবে না। এমনকি আপনি যে কোন সময় সাক্ষাৎকার বন্ধও করতে পারেন। কিন্তু এ বিষয়ে আপনার মতামত আমাদের কাছে অনেক** গুরুত্বপূর্ণ**, তাই** আমি **আশা করব আপনি উত্তরগুলো দিয়ে** আমাকে **সাহায্য করবেন।**

**এই গবেষণায় অংশ গ্রহনের মাধ্যমে আপনি** কোনো **ক্ষতির সম্মুখীন হবেন না। এখান থেকে আপনি** কোনো **সুবিধাও পাবেন না। কিন্তু আপনার অংশগ্রহন আমাদের গবেষণার বিষয়টি ভালভাবে বুঝতে সাহায্য করবে।**

যদি এই **গবেষণার** ব্যাপারে কোনো প্রশ্ন থাকে তাহলে আপনি ডাঃ জান্নাতুল ফেরদৌস কে ফোন করতে পারেন (মোবাইল নাম্বার - ০১৬২৭৯১৪৪৭৩). এই গবেষণাটি সি আই পি আর বি এথিক্যাল রিভিউ কমিটি অনুমোদন করেছেন। যদি আপনি মনে করেন এই **গবেষণায়** যোগ দিয়ে আপনি ঠিক ব্যবহার পাননি অথবা মনকষ্ট পেয়েছেন তাহলে আপনি ডাঃ মাহফুজুর রহমানকে ফোন করতে পারবেন। তার ফোন নাম্বার হচ্ছে- ০১৭৩০৯৩৮৭৪.

**যদি আপনার কোন প্রশ্ন থাকে তাহলে** আমি **উত্তর দিতে বাধিত হবো। আপনি যদি অনুমতি দেন তবে** আমি **শুরু করব।** আমি **কি শুরু করতে পারি?**

উত্তরদাতার স্বাক্ষরঃ

তারিখঃ

সময়ঃ

সাক্ষাৎকার গ্রহণকারীর স্বাক্ষরঃ

মৌখিক সম্মতিপত্র

৬ থেকে ১৫ বছর বয়সী শিশুর জন্য

**আমার নাম..................... । আমরা একটি গবেষণা পরিচালনা করছি এবং আমাদের গবেষণার উদ্দেশ্য হচ্ছে** ১৫ বছর এবং এর নিচের বয়সী শিশুদের চোখের সমস্যা এবং অন্ধত্ব এর হার এবং কারণগুলো খুঁজে বের করা এবং সেই পরিপ্রেক্ষিতে শিশু অন্ধত্ব প্রতিরোধমূলক ফ্রেম ওয়ার্ক প্রতিষ্ঠা করা। তোমাকে আমরা বাছাই করেছি কারণ তোমার বয়স ৬ থেকে ১৫ বছরের মধ্যে। এই গবেষণায় অংশগ্রহণের পূর্বে আমরা এটি নিশ্চিত করতে চাই যে তুমি আমাদের উদ্দেশ্য এবং তোমার কি করতে হবে তা সম্পর্কে ঠিকমতন বুঝতে পেরেছো। তুমি যদি আমার কোন কথা না বুঝতে পারো আমাকে জিজ্ঞেস করতে পারবে।

**আমি একটি টর্চ দিয়ে তোমার চোখ দেখবো এবং চার্ট এর মাধ্যমে তোমার চোখের তীক্ষ্ণতা পরীক্ষা করবো যার জন্য আমার ১৫-২০ মিনিট সময় লাগবে। তোমার চোখ পরীক্ষার ফলাফল গোপন রাখা হবে এবং গবেষণা দলের বাহিরে কারো সাথে এই বিষয়ে আলোচনা করা হবে না। তোমার ব্যক্তিগত তথ্যসমূহ গবেষণা ব্যতিত অন্য কোন কাজে ব্যবহার করা হবে না।**

**তোমার অংশগ্রহন হবে স্বেচ্ছা মূলক এবং তুমি কোনো প্রশ্নের উত্তর দিতে পছন্দ না করলে তোমাকে উত্তর দিতে হবে না। তুমি চাইলে যে কোন সময় সাক্ষাৎকারও বন্ধ করতে পারো। কিন্তু এ বিষয়ে তোমার অংশগ্রহন আমাদের কাছে অনেক** গুরুত্বপূর্ণ**, তাই** আমি **আশা করব এই ব্যাপারে তুমি** আমাদেরকে **সাহায্য করবে।**

**এই গবেষণায় অংশ গ্রহনের মাধ্যমে তুমি** কোনো **ক্ষতির সম্মুখীন হবে না। এখান থেকে তুমি** কোনো **সুবিধাও পাবে না। কিন্তু তোমার অংশগ্রহন আমাদের গবেষণার বিষয়টি ভালভাবে বুঝতে সাহায্য করবে।**

যদি এই **গবেষণার** ব্যাপারে কোনো প্রশ্ন থাকে তাহলে তুমি ডাঃ জান্নাতুল ফেরদৌস কে ফোন করতে পারো (মোবাইল নাম্বার - ০১৬২৭৯১৪৪৭৩)। এই গবেষণাটি সি আই পি আর বি এথিক্যাল রিভিউ কমিটি অনুমোদন করেছে। যদি তুমি মনে করো এই **গবেষণায়** যোগ দিয়ে তুমি ঠিক ব্যবহার পাওনি অথবা মনঃকষ্ট পেয়েছো তাহলে তুমি ডাঃ মাহফুজুর রহমান কে ফোন করতে পারো। তার ফোন নাম্বার হচ্ছে- ০১৭৩০৯৩৮৭৪।

**যদি তোমার কোন প্রশ্ন থাকে তাহলে** আমি **তার উত্তর দিতে বাধিত হবো। তুমি যদি অনুমতি দাও তবে** আমি **শুরু করবো।** আমি **কি শুরু করতে পারি?**

সাক্ষাৎকার গ্রহণকারীর নাম ও স্বাক্ষরঃ

তারিখঃ

সময়ঃ

**শিশুদের অন্ধত্ব এবং চোখের সমস্যা সম্পর্কীয় প্রশ্নাবলী**

| **মায়ের জন্য প্রশ্নাবলীঃ** | | | |
| --- | --- | --- | --- |
| **সি.না:** | **প্রশ্ন** | **উত্তর** | |
| ১) | আপনার প্রসবকালি সময়সীমা সাত মাস সম্পূর্ণ করেছিলো কি? | হ্যাঁ | না |
| **খালি চোখে এবং প্রযোজ্য টুল ব্যবহার করে শিশুর চোখ পরীক্ষাঃ** | |  | |
| ২) | আপাতদৃষ্টিতে শিশুর চোখের গঠনমূলক কোনো সমস্যা আছে কি?  সতর্কতাঃ পর্যাপ্ত আলোযুক্ত স্থানে শিশুর দুই চোখই দেখুন | হ্যাঁ | না |
| ৩) | শিশুর চোখের মনিতে কোনরকম সাদা দাগ/ দাগ আছে কি?  সতর্কতাঃ টর্চ এর মাধ্যমে শিশুর দুই চোখ দেখুন | হ্যাঁ | না |
| ৪) | শিশুর চোখের তি**ক্ষ্ণতা পরীক্ষাকরণ (নিচে বর্ণনা দেখুন)** | | |

প্রশ্ন ০4 এর জন্যঃ

| ছবি/ চার্টের মাধ্যমে শিশুর চোখের তি**ক্ষ্ণতা পরীক্ষাকরণ (বয়স উপযোগী টেকনিক)** | | | |
| --- | --- | --- | --- |
| **বয়স** | **টুল** | **প্যারামিটার** | |
|  |  | **শিশুর চোখের সমস্যা নেই** | **শিশুর চোখে সমস্যা আছে** |
| ১ বছর এবং এর নিচে | Follow and Fixation of Light | আলোর দিকে তাকায় এবং অনুসরণ করে | আলোর দিকেই তাকাচ্ছে না |
| ২ বছরের নিচে | Cake decoration test | রঙ্গীন মিষ্টির দিকে তাকাচ্ছে এবং হাতে তুলে নিচ্ছে | রঙ্গীন মিষ্টির দিকে তাকাচ্ছে না |
| ২ থেকে ৫ বছর | K- Picture chart | চার্ট এর আকৃতি এবং চিত্র পার্থক্য করতে সক্ষম হয়েছে | চার্ট এর আকৃতি এবং চিত্র পার্থক্য করতে সক্ষম হয়নি |
| ৬ থেকে ১৫ বছর | Snellen Chart | চার্ট এর চিনহিত লাইন পরতে পেরেছে | চার্ট এর চিনহিত লাইন পরতে পারেনি |
