# Supplementary material for: Epidemiology of childhood blindness: A community-based study in Bangladesh
Source: PLoS One. 2019 Jun 7;14(6):e0211991. doi: 10.1371/journal.pone.0211991 (PMC6555501; doi:10.1371/journal.pone.0211991)
Supplement: S2 File — (DOCX) [file pone.0211991.s002.docx]

**Informed written consent form**

**for**

**Parents/ caregivers of children**

My name is……… We are conducting a research and objective of our research is to find out the prevalence’s and causes of visual impairments and blindness among children aged ≤ 15 years and establishing a preventive framework for them in context of this problem. Your child has been selected as his/her age is within 0-15 years. We want to be sure that you understand the purpose and your responsibility in the research before you decide if you want to be in it. Please ask us to explain any words or information that you may not understand.

I want to ask you some questions regarding your child as well as test your child’s eye using a torch and examine his/her visual acuity with the help of a tool (torch, colorful sweeteners or chart) which will take 15-20 minutes. All of your answers and findings of the examinations will be kept confidential and will not be discussed with anyone outside the research team. Your personal information will not be used for any purpose other than research.

There is no physical, social or psychological risk in this study. You will not get any direct benefit from this study. You can avoid answering any question and stop the interview at any time if you do not feel comfortable. However, information from this study will help us to know the current scenario regarding this problem. So, that we can tackle the situation and improve the condition in your locality. Therefore, we are asking your voluntary participation in this regard.

If you have any questions about this research, you can call *Dr. Junnatul Ferdoush* *(Mobile- 01627914473)*. This research has been reviewed and approved by Centre for Injury Prevention and Research Bangladesh. If you have any questions about how you are being treated by the study or your rights as a participant, you may contact *Dr. Mahfuzar Rahman (Mobile- 01713093874).*

If you have any questions, I will be obliged to answer. I will start after getting permission from you.

Signature of the respondent:

Date:

Time:

Signature of the interviewer:

**Verbal consent form**

**for**

**Children aged 6-15 years**

My name is……… We are conducting a research and objective of our research is to find out the prevalence’s and causes of blindness and visual impairments among children aged ≤ 15 years and establishing a preventive framework for them in context of this problem. You have been selected as your age is within 6-15 years. We want to be sure that you understand the purpose and your responsibility in the research before you decide if you want to be in it. Please ask us to explain any words or information that you may not understand.

I want to see your eyes using a torch as well as examine your visual acuity with the help of a tool (torch, colorful sweeteners or chart) and it will take 15-20 minutes. The findings of your examination will be kept confidential and will not be discussed with anyone outside the research team. Your personal information will not be used for any purpose other than research.

There is no physical, social or psychological risk in this study. You will not get any direct benefit from this study. You can avoid answering any question and stop the interview at any time if you do not feel comfortable. However, information from this study will help us to know the current scenario regarding this problem. So, that we can tackle the situation and improve the condition in your locality. Therefore, we are asking your voluntary participation in this regard.

If you have any questions about this research, you can call *Dr. Junnatul Ferdoush* *(Mobile- 01627914473)*. This research has been reviewed and approved by Centre for Injury Prevention and Research Bangladesh. If you have any questions about how you are being treated by the study or your rights as a participant, you may contact *Dr. Mahfuzar Rahman (Mobile- 01713093874).*

If you have any questions, I will be obliged to answer. I will start after getting permission from you.

Name of the interviewer:

Date:

Time:

**Tool for childhood blindness & visual impairments**

**Pre-developed socio-demographic information sheet for the target group (Total Sample: 39351)**

| **Question to be asked to child’s mother:** | | | |
| --- | --- | --- | --- |
| **Sl. No.** | **Question** | **Response** | |
| 1) | Was your delivery period completed 7 months? | Yes | No |
| **Question (for mother) & examination part (for child) to be administered through data collector:** | | | |
| 2) | Have any visible deformities in child’s eyes?  *Instruction: Check both eyes of the child in a place with proper lighting.* | Yes | No |
| 3) | Have any kind of white spot/ spot in the pupils of the child’s eyes?  *Instruction: Examine both eyes of the child using torch.* | Yes | No |
| 4) | Testing of visual acuity of the child *(Instrument with parameter according to child’s age showed in the table below)* | | |

**Table for Q 4:**

| **Instrument with parameter to examine the visual acuity of the child (Age specific technique)** | | | |
| --- | --- | --- | --- |
| **Age of child** | **Instrument** | **Parameter used to screen cases** | |
|  |  | **Child has no visual problem** | **Child has visual problem** |
| ≤ 1year | Follow and Fixation of Light | Able to look and follow the light of the torchlight | Is not able to look |
| < 2years | Cake Decoration Test | Able to see and take the colorful sweeteners from white paper sheet | Is not able to see the colorful sweeteners |
| 2-5years | K- Picture Chart | Able to distinguish the shape/ identify the pictures in chart | Is not able to distinguish/ identify |
| 6-15years | Snellen Chart/ E-Chart | Able to read the cross mark in the chart | Is not able to read |
