# Supplementary material for: Epidemiology of childhood blindness: A community-based study in Bangladesh
Source: PLoS One. 2019 Jun 7;14(6):e0211991. doi: 10.1371/journal.pone.0211991 (PMC6555501; doi:10.1371/journal.pone.0211991)
Supplement: S3 File — (PDF) [file pone.0211991.s003.pdf]

চোখের আঘাত

Ocular Trauma

অভিভাবককে প্রশ্ন করুন

- আপনার সন্তানের চোখে কোন আঘাত লেগেছিল কি না?

Question to Guardians:

- Where your child's eyes injured?

টর্চ দিয়ে পরীক্ষা করুন

- চোখে আঘাতের কোন চিহ্ন আছে কি না?

Examine with a torch

- is there any evidence of any injury?

World Health Organization
Country Office for Bangladesh

চোখের ঘা (আলসার)

Corneal Ulcer

অভিভাবককে প্রশ্ন করুন

- আপনার সন্তানের চোখে কোন আঘাত লেগেছিল কি না?
- আলোতে তাকাতে অসুবিধা হয় কি না?

Question to Guardians:

- Where your child's eyes injured?
- Have trouble looking at light?

টর্চ দিয়ে পরীক্ষা করুন

- চোখের মণিতে সাদা দাগ দেখা যায় কি না?

Examine with a torch

- Have white spots in the pupil of the eyes?

World Health Organization
Country Office for Bangladesh

রেটিনোপ্যাথি অব প্রিম্যাচিউরিটি (ROP)

অভিভাবককে প্রশ্ন করুন

- আপনার বাচ্চা ৩৫ সপ্তাহ বা তার আগে জন্মগ্রহণ করেছে কি না?
- জন্মের সময় বাচ্চার ওজন ২ কিলোগ্রাম বা তার কম ছিল কি না?

Question to Guardians:

- Was your child born on 35 weeks or before?
- At the time of birth was the child's weight 2 kilogram or less?

World Health Organization
Country Office for Bangladesh

মস্তিষ্কের সমস্যাজনিত অন্ধত্ব

Cortical Visual Impairment (child with poor or no visual response)

অভিভাবককে প্রশ্ন করুন

- আপনার সন্তান স্বাভাবিকভাবে আপনার দিকে তাকায় কি না?

Question to Guardians:

- Does your child look at you normally?

World Health Organization
Country Office for Bangladesh

চোখের ছানি

Cataract

অভিভাবককে প্রশ্ন করুন

- আপনার সন্তানের চোখে দেখতে অসুবিধা হয় কি না?
- আপনার সন্তানের চোখের মণি সাদা বা ধূসর কি না?

Question to Guardians:

- Does your child have problem seeing?
- Is your child's pupil white or grey?

টর্চ দিয়ে পরীক্ষা করুন

- শিশুটির চোখের মণি সাদা বা ধূসর কি না?

Examine with a torch

Whether there are any white spot/ spot in the pupils of the child's eyes

World Health Organization
Country Office for Bangladesh

রেটিনোব্লাসটোমা
(এটি একটি চোখের ক্যান্সার)

Retinoblastoma

অভিভাবককে প্রশ্ন করুন
- শিশুটির চোখের মণি সাদা দেখা যায় কি না যা সাধারণত রাতে আলোতে বিড়ালের চোখের মত জ্বলজ্বল করে।

Question the Guardians:
- Does the childs pupil look white and do they generally glow at night like a cat.

টর্চ দিয়ে পরীক্ষা করুন
- সাদা চোখ আলোতে জ্বলজ্বল করে কি না?

Examine with a torch
- Does the white eye glow in light?

World Health Organization
Country Office for Bangladesh

ট্যারা চোখ

Squint

অভিভাবককে প্রশ্ন করুন

- আপনার সন্তানের চোখ বাঁকা কি না অথবা মাঝে মাঝে বাঁকা হয়ে যায় কি না?

Question to Guardians:

- Does your child have curved eyes or do they sometimes gets curved?

নিজে পর্যবেক্ষণ করুন

- চোখ কোনদিকে বাঁকা অথবা ট্যারা হয়ে আছে কি না?

Observe yourself

- Which way is the eye curved or is it squint?

World Health Organization
Country Office for Bangladesh

চোখের গঠনগত ত্রুটি

Congenital Anomaly of Eyes

অভিভাবককে প্রশ্ন করুন

- আপনার সন্তানের চোখে গঠনগত অস্বাভাবিকতা আছে কি না?

Question to Guardians:

- Is there any structural abnormality in your child's eyes?

নিজে পর্যবেক্ষণ করুন, শিশুটির

- চোখের পাতা নিচে নেমে এসেছে কি না?
- সামনের দিকে চোখ বের হয়ে আছে কি না?
- চোখের মণি অস্বাভাবিক বড় কি না?
- চোখ অস্বাভাবিক ছোট কি না?

Observe yourself, is the child's

- Eyelids coming down?
- Eyes are coming out towards the front?
- Cornea enlarged?
- Eyes abnormally small?

World Health Organization
Country Office for Bangladesh
